# VLUIS, a land use data product for Victoria, Australia, covering 2006 to 2013

**DOI:** 10.1038/sdata.2015.70

**Published:** 2015-11-24

**Authors:** Elizabeth Morse-McNabb, Kathryn Sheffield, Rob Clark, Hayden Lewis, Susan Robson, Don Cherry, Steve Williams

**Affiliations:** 1 Agriculture Research, Department of Economic Development, Jobs, Transport & Resources, Epsom, Victoria 3551, Australia; 2 Agriculture Research, Department of Economic Development, Jobs, Transport & Resources, Parkville, Victoria 3053, Australia; 3 Agriculture Research, Department of Economic Development, Jobs, Transport & Resources, Tatura, Victoria 3616, Australia; 4 Agriculture Research, Department of Economic Development, Jobs, Transport & Resources, Horsham, Victoria 3400, Australia

**Keywords:** Geography, Databases, Agriculture, Environmental social sciences

## Abstract

Land Use Information is a key dataset required to enable an understanding of the changing nature of our landscapes and the associated influences on natural resources and regional communities. The Victorian Land Use Information System (VLUIS) data product has been created within the State Government of Victoria to support land use assessments. The project began in 2007 using stakeholder engagement to establish product requirements such as format, classification, frequency and spatial resolution. Its genesis is significantly different to traditional methods, incorporating data from a range of jurisdictions to develop land use information designed for regular on-going creation and consistency. Covering the entire landmass of Victoria, the dataset separately describes land tenure, land use and land cover. These variables are co-registered to a common spatial base (cadastral parcels) across the state for the period 2006 to 2013; biennially for land tenure and land use, and annually for land cover. Data is produced as a spatial GIS feature class.

## Background & Summary

Land use mapping is conducted in Australia at two principal scales: national and state, and commonly utilises a combination of satellite imagery, agricultural census commodity statistics and other more local land use data. National-scale land use mapping is useful for baseline setting and evaluating regional development programs as it provides a continent-scale picture of land use activity at a coarse resolution. State-scale mapping in each of the Australian state and territory jurisdictions provides finer detail, supporting the development of policy and change monitoring programs at this level.

In the state of Victoria, contemporary, detailed state-wide land use mapping commenced in 1996 with full coverage completed by 2005. The process to produce that initial single map was both time consuming and labour intensive, requiring 9 years of field and office work. The 1996–2005 dataset utilised several sources of data, and different regions were surveyed independently, each employing slightly different methods that produced inconsistencies across the state. That dataset provided an essential resource for many research projects related to land use planning and catchment hydrology modelling. Value was still achieved, despite some of the early data recorded becoming out-dated before the dataset was fully completed in 2005.

In 2007 the Victorian Land Use Information System (VLUIS) project was established due to an ongoing need for reliable and consistent land use information. To ensure that the final product would benefit the greatest range of users, a ‘Land Use Summit’ was held in November 2007 to engage data users. 28 organisations were represented by 48 people at the event from 55 organisations that were invited to participate in the process. The organisations included local government authorities, agricultural industry groups, a wide range of state government organisations and three federal organisations. Prior to the event a survey was sent to all known users and creators of land use information in Victoria to gain a general understanding of the current status of relevant data. The summit built on these survey findings to determine the ideal spatial, temporal and contextual design of land use data. Results clearly showed that every user had differing needs and methods for analysing the data and that it would be difficult to design a system that achieves ‘everything for everyone’. Nevertheless, the fundamental requirements, consistent across all stakeholders, was for regular data updates, consistent classifications, and mapping to the smallest unit possible.

**Figure 1:**
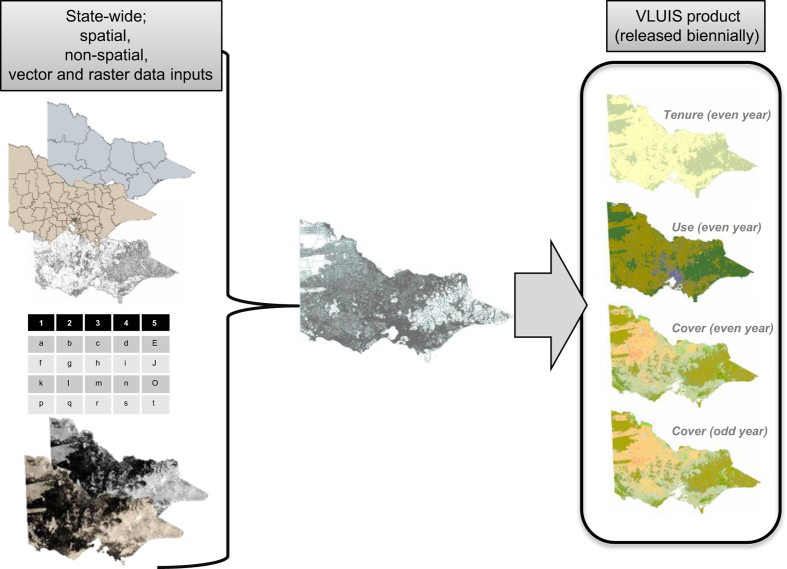
The VLUIS product is developed by integrating state-wide, spatial and non-spatial, vector and raster data inputs.

As a result, a new practical and efficient methodology for the generation and integration of land information data was created. This allows annual or biennial data renewal, and links the data directly to a common framework based on cadastral (or land) parcel units, which are the smallest unit of land ownership in Victoria. It also separately describes land tenure (ownership), land use (type of property) and land cover (surface cover type). Derived from three different sources, the dataset is an integrated combination of spatial and non-spatial, raster and vector data (Fig. 1). Foundation information sources include the Victorian Government Corporate Spatial Data Library (CSDL)^1,2^, the Valuer General Victoria (VGV) General Revaluation tables^3–5^ and land cover information generated from MODIS satellite imagery^6^.

Four state-wide spatial data products have been created to cover the years 2006/07, 2008/09, 2010/11 and 2012/13. Each is a vector dataset based on the state-wide cadastral parcel layer^1^. As well as land tenure, use and cover information, contextual information describing local government areas (LGAs) and Catchment Management Authority (CMA) regions is also attributed to each cadastral parcel to aid data analysis by stakeholders.

The structure and inherent fusion of data within the VLUIS data product enhances its potential use and reuse. The consistent data structure enables comparisons within and between years. Funding to produce the VLUIS data product, in the current structure, is secured until June 2017.

## Methods

A new VLUIS data product is released every second year. The land cover data is new data, created annually, whilst the land tenure and land use data are sourced biennially (Table 1). A single VLUIS data product is therefore comprised of land tenure information and land use information (for an even numbered year), and two sets of land cover information (one for an even numbered year and one for the following odd number year).

### Spatial Coverage and Context

**Figure 2:**
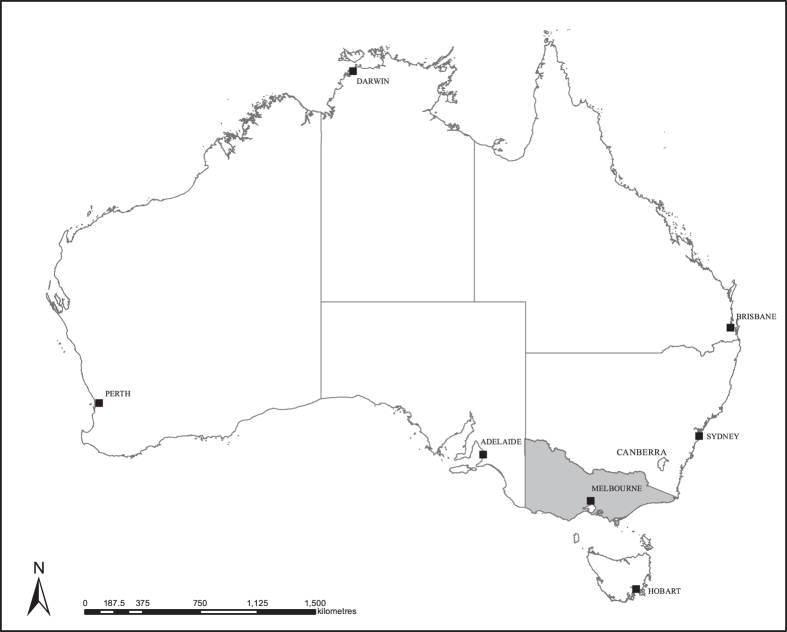
Victoria is situated in the south east corner of Australia. The land surface area is approximately 227,100 km^2^ (ref. 7).

The VLUIS data product is a state-wide land based dataset. The state of Victoria lies in the south-eastern portion of the Australian continent, bordered by New South Wales to the north and South Australia to the west. It has a land surface area of approximately 227,100 km^2^ with a southern coastline of approximately 1870 km (Fig. 2)^7^. With a population of around 5.7 million, the majority (70%) of which live in Melbourne, it is Australia’s most densely populated state. Approximately 30% of the state is contained within reserves and national parks and, other than the urban areas, diverse agricultural production dominates the remainder.

### Selection of Spatial Reference Unit

The final scale of the land use data was guided by user requirements to keep the spatial resolution as small as possible. The state cadastral parcel layer (V_PARCEL_MP) records the smallest unit of land ownership across the state^1^. Land ownership has a critical influence on the management and use of land and therefore provides a useful spatial context to land information. The state cadastre has several characteristics that make it useful as a basis for repetitive mapping, including:

- A unique state-wide parcel identifier that can be traced to a particular location.
- Consistency with local government area boundaries, and with well-defined tolerances.
- A well-established, effective and on-going maintenance program.

The VLUIS process takes the Permanent Feature Identifier (PARCEL_PFI), Standard Parcel Identifier (PARCEL_SPI) and the Local Government Authority (LGA) field from the dataset (V_PARCEL_MP) and these are the primary keys used to link all other datasets.

### Data used

#### *Land Tenure*

Public land Management (PLM25)^2^ information is compiled by State Government agencies and maintained via many datasets that detail the leasehold and management arrangements on public land. PLM25 has been used as the single source of land tenure information since 2010. Prior to that a series of smaller datasets were integrated and re-coded in VLUIS. Simple land tenure information (public or privately owned) is extracted from PLM25 and, as it is constantly updated, data is extracted at a date that matches the land use data.

#### *Land Use*

Local government rates for each property in Victoria are calculated using a state-wide general revaluation process every two years. This process is governed by the Valuer General Victoria (VGV) and is undertaken by valuation officers contracted by local government^3–5^. The valuation data records detail information about the property type and its buildings; infrastructure and location that is used to calculate the value of the land and improvements. The Australian Valuation Property Classification Code (AVPCC), or property type classifier, is synonymous with land use. A small proportion of the information collected by the valuations officers is available as ‘publicly releasable information’^8^. The ‘property type’ fits into this category and provides an accessible, consistent and detailed classification of land use. The property type (land use) data is provided in tabular form by VGV with each record containing an assessment number and an LGA based identifier. A unique key is created by concatenating these fields.

#### *Land Cover*

The VLUIS generates accurate state-wide land cover data on an annual basis using a remote sensing approach based on time series analysis of MODIS imagery^6,9–12^. MODIS imagery was chosen because it covers the entire state in one image, is free of charge, and has well documented, consistent products. The process of creating land cover data was developed within the VLUIS project. It has become a unique dataset in its own right and is developed using a lengthy process^13^.

### Classification used

Three classifications are used for the three main data sources. ‘Land tenure’ is described in a binomial classification that simply represents a land parcel as either public or private land in the VLUIS data (Table 2a). ‘Land use’ is classified using the AVPCC, which is the native classification of the VGV data^3–5^. The AVPCC scheme uses a hierarchical system, consisting of nine primary classes and many secondary, tertiary and quaternary classes (Table 2b). The ‘land cover’ classification system is also hierarchical. The primary level separates woody from non-woody vegetation and also water. The secondary level segments the woody and non-woody classes into five classes and the tertiary level has 12 classes in total (Table 2c). In all cases, the most detailed level of classification available is presented in the VLUIS data product.

### Overall method

Python scripting language has been used to automate components (LGA code, Zonal statistics, Public land identification) of the VLUIS output using ESRI’s ArcObjects through the geo-processing framework^14^. IDL scripting language^15^ was used to automate stages of the land cover creation process. Full automation is difficult due to the dynamic nature of the data sources.

**Figure 3:**
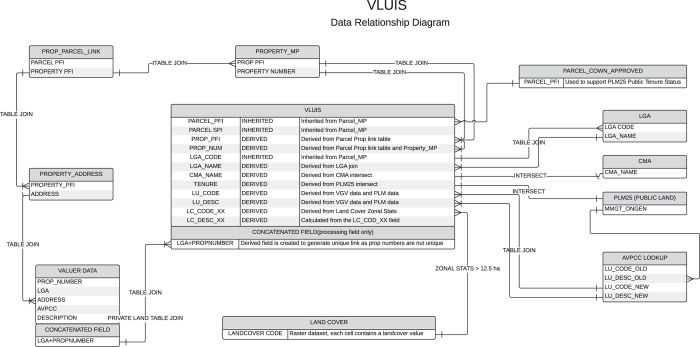
Overall data integration method used to create complete VLUIS product.

The VLUIS product utilises the state cadastral parcel layer (V_PARCEL_MP) as a spatial framework and source of a unique key for data integration. Land tenure is derived from PLM25 (or alternative tables prior to 2010) using the spatial intersection with PARCEL_PFI. This data is used as the definitive source of public land information. Once the public land areas are defined, information for the remaining areas is taken from VGV data. The VGV data provides excellent classifications of property type (land use) but is only available as a non-spatial table. Therefore a large effort is required to create unique, spatially attributed keys to link VGV data to the base cadastral layer. The process has many steps and requires numerous joining keys, as outlined in Fig. 3. As the land cover data is spatially continuous for the state, all parcels greater than 12.5 ha can be attributed. Those parcels smaller than 12.5 hectares are not attributed as the spatial resolution of the original MODIS imagery and classification system cannot support that scale. Finally, CMA regions are linked to PARCEL_PFI based on the region at the centroid of a parcel (Fig. 3).

## Data Records

All data required to create the VLUIS product are combined into one ESRI feature dataset^14^. There are 17 fields that are described below in Table 3.

The data products for each time period are available for download from a number of locations. The data has been loaded onto the data repository ‘Dryad’(Data Citation 1) as ESRI shapefiles. The data is also available as ESRI feature data products from the Victorian Government Data Directory (https://www.data.vic.gov.au/data/dataset/victorian-land-use-information-system-2006-2007, 2008–2009 or 2010–2011 or 2012–2013). This data repository includes a register and tools to freely access the dataset. In addition, the VLUIS data can be accessed as a zip file through the Australian Collaborative Land Use and Management Program (ACLUMP) website (http://www.daff.gov.au/ABARES/aclump/Pages/land-use/data-download.aspx) which has the additional fields shown in Table 4 to align the VLUIS product with mapping undertaken in other Australian states and territories.

## Technical Validation

The primary accuracy assessment of the land tenure and land use data resides with the original custodian, however, in some cases a further assessment of the accuracy of the data within the VLUIS product has been undertaken and are described below.

The process of maintaining land tenure data in Victoria is well documented and consistent^2^. It is difficult to determine tenure visually ‘on-ground’ and it rarely changes, therefore it was considered unnecessary to undertake an independent validation of the data.

Land use data from the VGV has three consistency checks, firstly by the VGV when the data comes in from local governments across the state; secondly when it is released back to local government, and thirdly by individual review through the land tax process. However; the VGV data is used in a unique way in the VLUIS process and therefore a small set of independent checks have been made on the ‘spatial’ VLUIS representation of the data for each product. Where available; independent data sources that map land use for industry or commercial purposes have been used to check the VGV data. However, there are issues with most data sources of this type, as they commonly map localised regions, represent only one point in time, are not maintained and may be point-based or have low spatial accuracy. The most useful source of accuracy assessment for the land use data component has been to release the data in draft form as soon as possible and encourage feedback from data users. The 2008/09 and 2010/11 data were released to 72 individuals from a wide range of organisations before open publication (Table 5).

As land cover data was generated for the VLUIS product the validation data collection and accuracy assessment was undertaken within the project. Each year between October and December, from 2009 to 2013, extensive field campaigns were undertaken for assessments of land cover type. Field assessments are taken in a stratified random clustered approach and note the type of land cover present. The information is used for calibration of the MODIS satellite imagery and validation of the resultant Tertiary Dominant Land Cover (TDLC) product. In 2012, financial and resource constraints meant that fewer calibration and validation data were collected. The land cover data from 2009–2013 has been created using 50% of the ground data as calibration only, 25% as an interim validation check and the remaining 25% as validation only through generation of error matrix and total error estimates (Table 6)^13^.

There was no field data collection in 2006, 2007 and 2008, providing no means to create a TDLC product for these years. Therefore 2006, 2007 and 2008, land cover attribution in the VLUIS product is at the secondary level only, as this level (and the primary level) can be created without calibration data in any year. Similar SDLC and PDLC land cover classifications were made in 2009 to 2013 and the overall accuracies for these products are shown in Table 5 (above). As the same method is used across all years (2006 to 2013) it may be assumed that the accuracies of the 2006, 2007 and 2008 products are also at least 70% for the SDLC and 90% for the PDLC.

Once all data is created and integrated to form the VLUIS data product it undergoes a consistency check by the development team, which is then used to improve the immediate data outputs and highlight future data issues such as seasonal variation effects. This check utilises the hierarchical nature of the three land information levels (tenure, use and cover) to identify any potentially erroneous classifications through a series of logical definition queries in ArcGIS^14^. The process also ensures that all parcels have a classification at all levels and that the codes and descriptions are consistent. Finally, a visual check of the data is undertaken, which can highlight issues and errors not detectable by tabular logic methods.

## Usage Notes

As the VLUIS product is a complex and large volume dataset, usage and visualisation information is provided with the data release. However, being a fundamental dataset its applications are myriad and future uses may be unanticipated. In this setting it is difficult to support all potential usage, and as a consequence more effort is focused on description of the data and its qualities. With this aim, standard metadata records have been supplemented with more verbose usage information and as there are many land use and land cover classes, a classification symbology layer is also provided to facilitate data visualisation^16^.

## Additional Information

**How to cite this article**: Morse-McNabb, E. *et al.* VLUIS, a land use data product for Victoria, Australia, covering 2006 to 2013. Sci. Data 2:150070 doi: 10.1038/sdata.2015.70 (2015).

## Supplementary Material



## Figures and Tables

**Table 1:** List of data required to create VLUIS product.

**Input data**	**Variables**	**Source**	**Spatio-temporal resolution**
Victorian Cadastral Parcels (V_PARCEL_MP)	Dimensions and unique identifier numbers.	Vic Gov^1^	Biennial data in spatial format
Land tenure (PLM25)	Ownership and management on public land	Vic Gov^2^	Regularly updated in spatial format
Property valuation	Australian Valuation Property Classification Code	Valuer General Victoria^3–5^	Biennial data in tabular format
MODIS	EVI, NDVI, Red, NIR, and data quality bands.	NASA^6^	16 day composite of daily imagery for a full calendar year (MOD13Q1)
Local Government Areas (LGA_POLYGON)	LGA code and name	Vic Gov^1^	Updated when required
Catchment Management Authority Areas (CMA_100)	CMA name	Vic Gov^1^	Updated when required
Property Address (ADDRESS)	Property permanent feature identifier (PROP_PFI) and Address	Vic Gov^1^	Continuously updated
Primary, Secondary and Tertiary Land Cover (PDLC, SDLC, TDLC)	Land cover code and description	Created for VLUIS product^13^	Annual data in raster format
Property to Parcel linking table (PROP_PARCEL_LINK)	Parcel and parcel permanent feature identifier (PARCEL_PFI, PROP_PFI)	Vic Gov^1^	Continuously updated

**Table 2:** Three separate classification systems are used for the three separate datasets.

**(a) Land Tenure**	
2 classes	PrivatePublic
**(b) Land Use (AVPCC)**	
Primary Land Use 9 classes	ResidentialCommercialIndustrialExtractive IndustriesPrimary ProductionInfrastructure and UtilitiesCommunity ServicesSport Heritage and CultureConservation
Secondary Land Use Tertiary Land Use Quaternary Land Use	71 classes578 classes167 classes^4^
**(c) Land Cover**	
Primary Dominant Land Cover	UnknownWaterWoody vegetationNon-woody vegetation
Secondary Dominant Land Cover	UnknownWaterNative woody coverWoody vegetation - productionPasture and grasslandNon-woody vegetation - production
Tertiary Dominant Land Cover 12 classes and an Unknown category Land Cover classifications for parcels smaller than 12.5 ha are set as Not Applicable	UnknownWaterNative woody coverDeciduous woody horticultureEvergreen woody horticultureHardwood plantationSoftwood plantationPasture and grasslandBrassicasCerealsLegumesNon-woody horticultureBare and non-photosynthetic material

**Table 3:** Field names and descriptions found in the final VLUIS product.

**Field Name**	**Source**	**Field Description**
Parcel_PFI	PARCEL_MP	Parcel Persistent Feature Identifier is a unique number across the state that represents a particular parcel.
Parcel_SPI	PARCEL_MP	Parcel Standard Parcel Identifier is another unique identifier created from the concatenation of the ‘Lot’ number and ‘Plan’ number
Propnum	PROPERTY_MP	LGA Property Number
Prop_PFI	PROPERTY_MP	Property Persistent Feature Identifier is a unique number across the state for each property
LGA CODE	LGA	Each LGA across the state has a code
LGA name	LGA	Name of LGA
TENURE	PLM25	Classed as either public or private
LU_CODE	VGV & PLM25	Four digit code described in the AVPCC
LU_DESC	VGV & PLM25	Full text land use description that links with the LU_CODE
LU_1	VGV & PLM25	Single digit number that identifies the primary AVPCC class
LU_2	VGV & PLM25	Double digit code that identifies the secondary AVPCC class
LU_3	VGV & PLM25	Three digit code that identifies the tertiary AVPCC class
LC_CODE	LAND COVER	One to three digit code for each land cover type
LC_DESC	LAND COVER	Full text description of each dominant land cover type
CMA	CMA	CMA name
Shape_Length	PARCEL_MP	Parcel shape length
Shape_Area	PARCEL_MP	Parcel shape area in m^2^

**Table 4:** Additional data fields attributed by ACLUMP.

**Field Name**	**Field Description**
ALUM_CODE	Numerical Australian Land Use and Management (ALUM) Classification Version 7 code
LU_CODEV7	Australian Land Use and Management (ALUM) Classification Version 7 code
TERTIARY_V7	Australian Land Use and Management (ALUM) Classification Version 7 tertiary level description.
SECONDARY_V7	Australian Land Use and Management (ALUM) Classification Version 7 secondary level description
PRIMARY_V7	Australian Land Use and Management (ALUM) Classification Version 7 primary level description

**Table 5:** Data supplies to organisational groups prior to public release.

**Organisation type**	**Number of individual data supplies**
Internal (same organisation as development)	31
Wider State Government	18
Catchment Management Authority	8
National organisation (Australia)	8
Private consultant	10
University	6
International	2

**Table 6:** The number of observations made in each year from 2009 to 2013 and the resultant overall accuracy of the Tertiary Dominant Land Cover (TDLC), Secondary Dominant Land Cover (SDLC) and Primary Dominant Land Cover (PDLC).

	**2009**	**2010**	**2011**	**2012**	**2013**
Total number of field observations	4681	4989	4470	2839	5985
Number of observations used in error matrix	1160	1237	1109	1246	1485
TDLC overall accuracy	68.28%	64.51%	64.47%	61.64%	66.06%
SDLC overall accuracy	72.88%	77.36%	74.52%	80.86%	76.35%
PDLC overall accuracy	91.02%	91.02%	94.31%	96.75%	92.87%
